# Arrest of Hemorrhage in Placenta-Previa

**Published:** 1874-02

**Authors:** J. Matthews Duncan


Mechanism of Arrest of Hemorrhage in Placenta Prævia.—*J. Matthews Duncan, M.D.*—The restraint or absolute stoppage of hemorrhage in cases of placenta prævia may be produced in a variety of ways. The flow of blood through tubes of a peculiar shape is a pure mechanical phenomenon, and its arrest is also such. * * * The coagulation of blood in the maternal cells, or blood-spaces of the separated portion of placenta, is well known and recognized as the cause of the stoppage of the flow of blood-through them from the corresponding cells of the still attached portion. * * * In the so-called circular or marginal sinus of the placenta, I have never observed such a clot, as would form anything like a complete obstruction to the passage of blood along the course of the vessel. Bleeding through this circular sinus may be arrested by complete separation of the placenta, or by thrombosis of all those portions of placenta from which the open sinus gets its supplies of maternal blood.

In regard to the uterine sinuses, I am not aware of any observations which would lead us to suppose that clots of blood in their course are formed during labor, at their orrifices; it is probable that clots are formed during labor, which may enter them, cork-like, for a very short distance; such cork-like clots may form an obstacle to the flow of blood from them, but I do not believe their resisting force is so great as to be ever of much consideration in the stoppage of bleeding.

There is a general consent, and I believe justly so, that the divided curling arteries are not a source of ordinary uterine hemorrhage, whether unavoidable or accidental.

When the placenta is partially separated, the uterine sinuses corresponding to the detached portion will naturally be expected to collapse on account of the failure of the current of blood, from the now unconnected portion of the placenta, which formerly kept them more or less distended. * * * It is in this way—namely, by collapse and change of shape—that the bleeding is arrested in cases of placenta prævia, while uterine action is absent and whether labor is going on or not. * * * In the same way is the arrest of bleeding accounted for, in cases of successful resort to Simpson's or Barnes's method of treatment, by total or partial separation of the placenta; for in such cases there is not necessarily uterine action, but only detachment and stoppage of the ordinary course of the circulation of the whole or of a portion of placenta in this region. The arrest of the bleeding in all cases of separation of the placenta, when the uterus is not in action, is to be explained in the same manner.

Uterine action will increase the change of shape favorable to the arrestment of bleeding, by compressing the uterine wall and its contained vessels between its own contracting tissue and the separated portion of placenta or unruptured sac of the ovum, or the adjacent fœtal part. We do not say that such compression will stop the hemorrhage flowing from the uterine sinuses; but it may do so, and it will certainly help towards this end. Pains, or mere uterine action, have been very generally supposed to be a cause of hemorrhage, but it is plain that they cannot be so directly, as I have elsewhere attempted to show.

In cases of placenta prævia, there are frequently only moderate or even small gushes of blood, both before and after labor commences. It is both natural and proper to regard these bleedings as being the result of emptying of replete vessels, and as stopped by the production of local anæmia. In great bleedings, general anæmia will be rapidly produced; and such general anæmia, being accompanied by great diminution of blood pressure, will certainly lead to diminution or even arrest of flooding. * * * The adspiratory or sucking-in force in the abdomen cannot prevent hemorrhage while the placenta is being detached; but other hemorrhage, in connection with placenta prævia, it may not only restrain or stop, but it may more truly and frequently be described as preventing.

It is impossible to contemplate the sources of bleeding in placenta prævia without recognizing the beneficial influence of pressure, however exerted, on the bleeding parts. Authors greatly mistake in regarding the cervix as the part to be pressed. That is not the bleeding part. They also mistake in regarding the lowest or presenting part of the child as the part pressing. The part of the ovum pressing, or that may press, the bleeding surface, is the presenting part of the child only in very early labor, and then, in most cases, only after rupture of the membranes.

Spontaneous complete separation of the placenta, when it is prævia, is frequently accompanied or followed by arrest of hemorrhage, frequently by diminution of its amount. The frequency of arrest of the bleeding by spontaneous separation of the placenta has been very much exaggerated by the enthusiastic Simpson. Nothing can be more unsatisfactory than the character of the statistical data on which he founds his opinion, that "in one only out of every twenty-two labors does there appear to have been a continuation of hemorrhage to a great or profuse degree after the placenta was detached;" and the statement is, in my opinion, of little value. * * * In forty-seven cases, where there was a sufficient interval between the separation of the placenta and expulsion of the child, to judge whether bleeding was stopped or not, I find that in twenty-five cases only, or in a little more than one in two, was the bleeding arrested. In all the rest it appears to have continued in greater or less degree. * * * In cases in which complete spontaneous separation of the placenta takes place, and in which the bleeding is arrested, it has to be asked, Is the stoppage of the bleeding the consequence of the complete separation of the placenta? I can see no reason whatever for thinking so. * * * But, if the frequent arrest of the bleeding after complete separation is not accounted for by the detachment of the placenta, how is it explained? * * * Spontaneous separation is indisputable evidence of energy, of labor, and of great progress of the first stage. In short, complete spontaneous separation of the placenta indicates such characters in a labor as would lead us to expect early arrest of the bleeding, apart entirely from the remarkable circumstance of the complete separation.

In itself, complete separation of the placenta must have an injurious tendency, if there is any truth in the views universally entertained as to the source of the hemorrhage; for it increases the bleeding area to the greatest possible extent. Artificial premature detachment of the whole placenta I shall not here discuss, because, with some change, most of the remarks made on the spontaneous separation apply to it as well.

It is a great mistake to suppose that "the one constant condition" of arrest of bleeding, in any circumstances, "is contraction, active or tonic, of the muscular structure of the uterus." Very many cases, indeed, of arrest of bleeding occur quite independently of uterine action. * * * The evacuation of the liquor amnii, whether spontaneous or artificial, is a great means of contributing to this progressive uterine shrinking, and this is the chief part of the explanation of its hemostatic influence; but it may also contribute to hemostasis indirectly, by increasing active uterine action or pains. The evacuation of liquor amnii, then, and the advance of labor, both offer ever increasing security against the persistence or recurrence of hemorrhage. Eminent obstetricians have tried to unsettle the professional faith in a contracted uterus as an efficient hemostatic after delivery. * * * The proposition I enunciate is, that it has never been shown that uterine hemorrhage, of the ordinary post-partum kind, ever takes place from the uterus when it is in a state of moderately firm general contraction. * * * Some uteri are extremely small (and, no doubt, proportionally weak) after delivery, scarcely larger than the fist; and such a uterus, if expanded to an ordinary size, would certainly be in a state of relaxation. If the uterus has sunk into the true pelvis, it may appear small and contracted when it is merely collapsed and depressed in the abdomen. An uterus may be hard and feel as if contracted, when the hardness is merely the result of passive tension. The bladder of urine and ovarian cysts frequently illustrate hardness of flaccid bags produced by passive distension. Great and even fatal hemorrhage may be erroneously supposed to be ordinary post-partum flooding, when it is really running from an unsuspected source.

